# Comparison of the Micellar Incorporation and the Intestinal Cell Uptake of Cholecalciferol, 25-Hydroxycholecalciferol and 1-α-Hydroxycholecalciferol

**DOI:** 10.3390/nu9101152

**Published:** 2017-10-23

**Authors:** Charles Desmarchelier, Marielle Margier, Damien P. Prévéraud, Marion Nowicki, Véronique Rosilio, Patrick Borel, Emmanuelle Reboul

**Affiliations:** 1 NORT Nutrition, Obesity and Risk of Thrombosis, Aix-Marseille University, INRA, INSERM, 13385 Marseille, France; charles.desmarchelier@univ-amu.fr (C.D.); marielle.margier@univ-amu.fr (M.M.); Marion.Nowicki@univ-amu.fr (M.N.); patrick.borel@univ-amu.fr (P.B.); 2 Adisseo France S.A.S., Center of Expertise and Research in Nutrition, 03600 Commentry, France; Damien.Preveraud@adisseo.com; 3 Institut Galien Paris Sud, UMR 8612, Univ Paris-Sud, CNRS, Université Paris-Saclay, 5 rue J.B. Clément, F-92296 Châtenay-Malabry, France; veronique.rosilio@u-psud.fr

**Keywords:** vitamin D, SR-BI, NPC1L1, ASBT, bioavailability, absorption, micelles, Caco-2 cells

## Abstract

In the context of the global prevalence of vitamin D insufficiency, we compared two key determinants of the bioavailability of 3 vitamin D forms with significant biopotencies: cholecalciferol, 25-hydroxycholecalciferol and 1-α-hydroxycholecalciferol. To this aim, we studied their incorporation into synthetic mixed micelles and their uptake by intestinal cells in culture. Our results show that 1-α-hydroxycholecalciferol was significantly more solubilized into mixed micelles compared to the other forms (1.6-fold and 2.9-fold improvement compared to cholecalciferol and 25-hydroxycholecalciferol, respectively). In Caco-2 TC7 cells, the hydroxylated forms were taken up more efficiently than cholecalciferol (*p* < 0.05), and conversely to cholecalciferol, their uptake was neither SR-BI(Scavenger-Receptor class B type I)- nor NPC1L1 (NPC1 like intracellular cholesterol transporter 1)-dependent. Besides, the apical membrane sodium–bile acid transporter ASBT (Apical Sodium-dependent Bile acid Transporter) was not involved, at least in vitro, in the uptake of any of the three vitamin D forms. Further investigations are needed to identify the uptake pathways of both 1-α-hydroxycholecalciferol and 25-hydroxycholecalciferol. However, considering its high bioavailability, our results suggest the potential interest of using 1-α-hydroxycholecalciferol in the treatment of severe vitamin D deficiency.

## 1. Introduction

First known as a regulator of phosphocalcic homeostasis, vitamin D has also been shown to play key roles in infectious, inflammatory, and metabolic diseases [1]. As most people get inadequate sun exposure to obtain or maintain a sufficient vitamin D status, they have to consume vitamin D, either as part of their diet or as supplements [2]. The current US recommended dietary allowance for vitamin D is 15 µg/day for healthy adults [3]. This target is very difficult to reach, given how few foods contain vitamin D [4], and although debated [5], the prevalence of vitamin D insufficiency is generally acknowledged globally [6,7].

As other lipophilic nutrients, cholecalciferol (vitamin D_3_, D_3_) has to be released from its food matrix to be incorporated into emulsified lipid droplets in the stomach and duodenum during the digestion process. D_3_ then transfers into mixed micelles (soluble particles constituted of phospholipids, cholesterol, lipid digestion products, and bile salts) and is transported in its micellar form to the brush border membrane of the enterocyte [8]. The apical membrane proteins SR-BI (Scavenger Receptor class B type I), CD36 (Cluster-Determinant 36), and to a lesser extent NPC1L1 (NPC1 like intracellular cholesterol transporter 1) have been involved in its intestinal uptake at dietary doses, whereas passive diffusion has been shown to predominate at supranutritional doses [9]. It must be noted here that the transfer of D_3_ throughout all these steps is only partial, and as a consequence, D_3_ bioavailability has been shown to range between 55% and 99% in healthy subjects [10] and displays very high interindividual variability [11]. 

**Figure 1:**
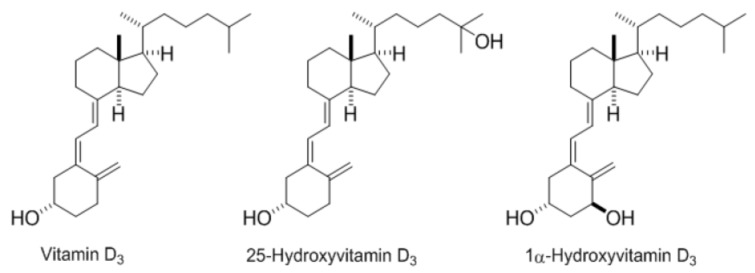
Vitamin D_3_ forms.

Beyond D_3_, other forms such as 25-hydroxyvitamin D_3_ (25(OH)D_3_) and 1alpha-hydroxyvitamin D_3_ (1α(OH)D_3_) are commercially available for supplementation and are already used in animal feedstuffs (Figure 1). Several animal studies reported differences in biopotency of these three molecules based on performance (feed efficiency, breast yield), physiological parameters (plasma 25(OH)D_3_, calcium and phosphorus concentration, bone mineralization), and/or incidence of specific disorders (rickets, tibial dyschondroplasia, osteomalacia) [12,13,14,15]. A review of these studies yielded the following biopotency ranking: 1α(OH)D_3_ > 25(OH)D_3_ > D_3_.

To investigate whether these differences in biopotency can be explained by difference in bioavailabilities, we performed in vitro studies with D_3_, 25(OH)D_3_, and 1α(OH)D_3_ to assess and compare (i) their solubilization in mixed micelles and (ii) their uptake by a model of human enterocytes, which are two key determinants of bioavailability; as well as (iii) the involvement of the membrane proteins SR-BI, NPC1L1, and ASBT (Apical Sodium–dependent Bile acid Transporter) in their uptake.

## 2. Materials and Methods

### 2.1. Chemicals

D_3_, ergocalciferol (vitamin D_2_), 25(OH)D_3_, 1α(OH)D_3_, retinyl acetate, 2-oleoyl-1-palmitoyl-sn-glycero-3-phosphocholine (phosphatidylcholine), 1-palmitoyl-sn-glycero-3-phosphocholine (lysophosphatidylcholine), monoolein, free cholesterol, oleic acid, sodium taurocholate, and simvastatin were purchased from Sigma-Aldrich (Saint-Quentin-Fallavier, France). Ezetimibe beta-d-glucuronide was purchased from Sequoia Research (Pangbourne, UK). Block lipid transport-1 (BLT1) was purchased from ChemBridge (San Diego, CA, USA). Dulbecco’s modified Eagle’s medium (DMEM) containing 4.5 g/L glucose and trypsin-EDTA (500 mg/L and 200 mg/L, respectively), non-essential amino acids, penicillin/streptomycin and PBS were purchased from Life Technologies (Illkirch, France), and fetal bovine serum (FBS) came from PAA (Vélizy Villacoublay, France). ASBT tagged with the DDK peptide (DYKDDDDK flag peptide) in pCMV plasmid and DDK antibody were purchased from Origen (Austin, TX, USA). All solvents used were HPLC grade (Carlo Erba Réactifs-SdS, Val de Reuil, France).

### 2.2. Preparation of Mixed Micelles for Micellar Incorporation Experiments and Cell Culture

Mixed micelles were formed as previously described [16,17]. Briefly, monoolein (0.3 mM), oleic acid (0.5 mM), phosphatidylcholine (0.04 mM), lysophosphatidylcholine (0.16 mM), and cholesterol (0.1 mM) dissolved in trichloromethane/methanol (2:1, *v*/*v*) and vitamin D forms (concentration range: 0–20 µM) dissolved in ethanol were transferred to a glass tube, and the solvent mixture was carefully evaporated under nitrogen. The dried residue was dispersed in DMEM containing 5 mM sodium taurocholate and was incubated at 37 °C for 30 min. The solution was mixed by sonication in a bath sonicator (Branson 3510 MT, 40 kHz; Branson Ultrasonics, Danbury, CT, USA) for 30 min and then incubated at 37 °C for 1 h. It was then filtrated through cellulose ester membranes (0.22 µm) (Millipore S.A.S., Molsheim, France), and the resulting solution of vitamin D-rich mixed micelles was stored at −20 °C until cell culture experiment or vitamin extraction and HPLC analysis. Concentrations of the different forms in the micellar solutions were checked before each experiment.

### 2.3. Cell Culture

#### 2.3.1. Caco-2 Cell Culture and Experiments

Caco-2 clone TC-7 cells were cultured as previously described [18,19]. For each experiment, cells were seeded and grown on Millicell^®^ hanging cell culture inserts (Millipore S.A.S., Molsheim, France) for 21 days to obtain confluent and highly differentiated cell monolayers. Twelve hours prior to each experiment, the medium in both the apical and basolateral chambers was replaced with serum-free complete medium. The apical uptake of vitamin D forms incorporated in mixed micelles was determined after a 1 h-incubation period in the presence or absence of inhibitors, as previously described [18,19]. The % of vitamin D taken up was estimated as the quantity of vitamin D present in the harvested cells divided by the sum of the quantity of vitamin D remaining in the apical chamber and that present in the harvested cells. The efflux of vitamin D forms incorporated in mixed micelles was determined as previously described [9], with minor changes. Briefly, cells first received the vitamin D-rich micelles at the apical side for 4 h. They were then washed once with PBS and equilibrated in serum-free complete medium for 15 min. Cells were either harvested (control) or they received apical medium containing vitamin D-free mixed micelles for 1 h. The % of vitamin D effluxed back to the apical side was estimated as the quantity of vitamin D present in the apical chamber divided by the quantity of vitamin D present in the harvested cells at t0.

#### 2.3.2. Griptite Cell Culture and Experiments

Griptite cells were cultured and transfected with 3 µg DNA (either ASBT in pCMV plasmid or empty pCMV control plasmid) similarly to HEK cells, as previously described [20,21]. Transfection efficiency was verified by western blotting, as previously published [22]. A pre-incubation of the cell monolayers with DMEM supplemented with either DMSO (control) or 100 µM simvastatin was performed during 30 min. Synthetic mixed micelles containing vitamin D forms were diluted in DMEM (1:5) to avoid cytotoxicity [23](data not shown). Apical uptake of vitamin D forms was determined after a 1 h-incubation with a solution of micellar vitamin D containing either DMSO or 100 µM simvastatin (final concentration of DMSO in both conditions: 0.01%) [20,21]. The % of vitamin D taken up was estimated as the quantity of vitamin D present in the harvested cells divided by the sum of the quantity of vitamin D remaining in the apical chamber and that present in the harvested cells.

All samples (harvested cells and culture medium after incubation) were sealed under nitrogen and stored at −80 °C until vitamin extraction and HPLC analysis.

### 2.4. Vitamin D Form Extraction

Vitamin D forms were extracted from 500 µL aqueous samples using the method previously described [9]. The internal standard was retinyl acetate for D_3_ and ergocalciferol for both 25(OH)D_3_ and 1α(OH)D_3_. After lipid extraction with hexane, dried residues were dissolved in 200 µL of mobile phase (acetonitrile/methanol/water, 60/38/2, *v*/*v*/*v*). A volume of 160 µL was used for HPLC analysis.

### 2.5. HPLC Analysis

The HPLC system and method were set up according to previous studies [9,18]. All molecules were identified by retention time compared with pure standards.

### 2.6. Statistical Analysis

Data were expressed as means ± SEM. Differences in Vitamin D form micellar incorporation and cellular uptake and efflux were tested using ANOVA (fixed-effects models). Prior to ANOVA, data were tested for equality of variances. Tukey’s test, which maintains the family-wise error rate at alpha = 0.05, was used as a post hoc test for pairwise comparisons. Student’s *t*-test was used to test the effect of membrane protein inhibition on the different form uptake. Data were tested for equality of variances and in case of inhomogeneous variances, Welch’s correction was applied to Student’s *t*-test. Values of *p* < 0.05 were considered significant. Statistical analyses were performed using SPSS (version 20, SPSS Inc., Chicago, IL, USA).

## 3. Results

### 3.1. Incorporation of the Different Vitamin D Forms in Synthetic Mixed Micelles

**Figure 2:**
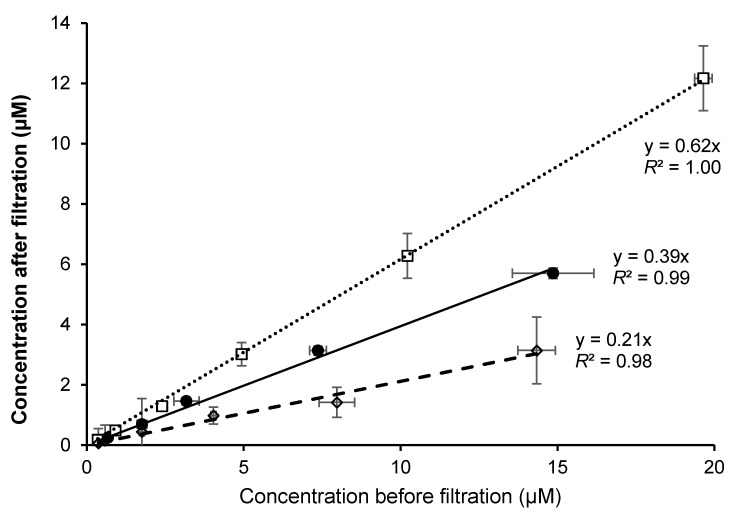
Incorporation of different forms of vitamin D in synthetic mixed micelles. Synthetic mixed micelles with varying concentrations of vitamin D forms were synthesized and the concentration of the different forms was measured by HPLC. Symbols: ●, D_3_; □, 1α (OH)D_3_; ◊, 25(OH)D_3_. Values are mean (*n* = 3) with their standard errors.

Incorporation curves of the three forms were linear and were significantly different (*p* = 0.03): 1α(OH)D_3_ was incorporated more efficiently (62%) than D_3_ (39%) and 25(OH)D_3_ (21%) (Figure 2).

### 3.2. Vitamin D Form Uptake by Caco-2 Cells

**Figure 3:**
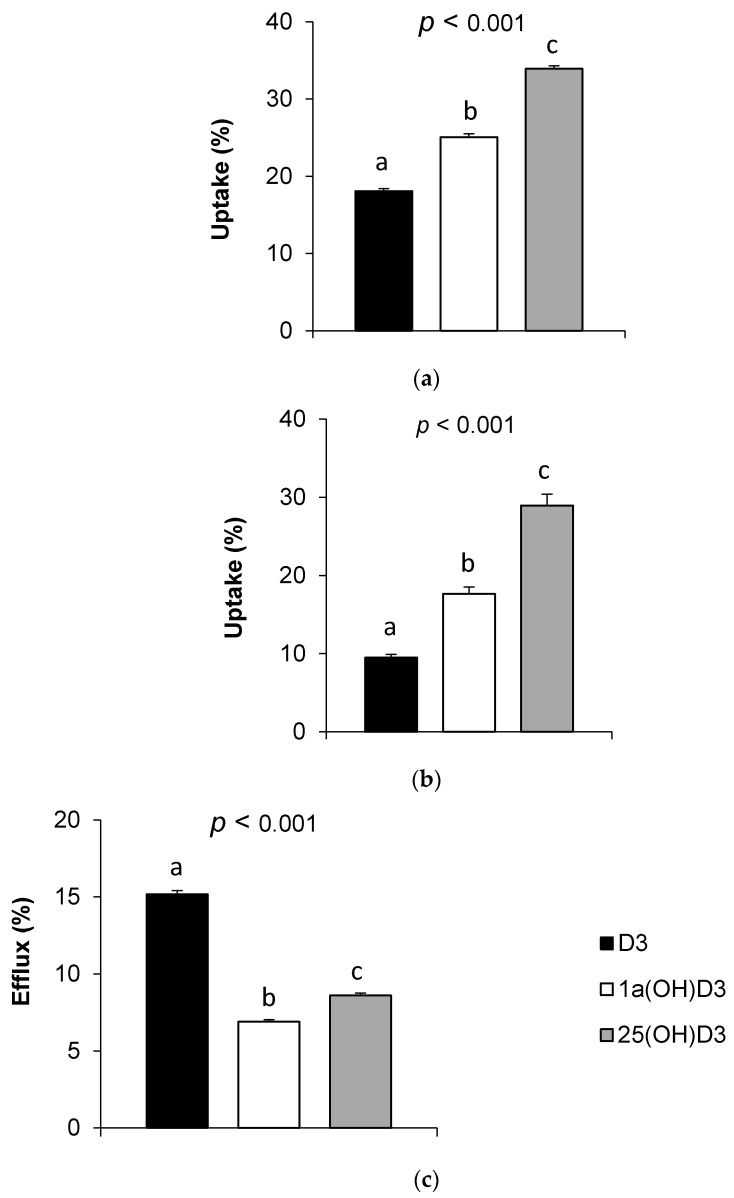
Uptake and efflux of vitamin D forms by Caco-2 cells. (**a**) Uptake of D_3_, 1α(OH)D_3_, and 25(OH)D_3_ at high concentrations, i.e., >5 µM. D_3_, 1α(OH)D_3_ and 25 (OH)D_3_ apical concentrations at t0 were 9.54, 11.17, and 7.22 µM respectively (*n* = 6); (**b**) Uptake of D_3_, 1α(OH)D_3_, and 25(OH)D_3_ at low concentrations, i.e., <1 µM. D_3_, 1α(OH)D_3_, and 25 (OH)D_3_ apical concentrations at t0 were 0.20, 0.37, and 0.10 µM, respectively (*n* = 3); (**c**) Efflux of D_3_, 1α(OH)D_3_, and 25(OH)D_3._ D_3_, 1α(OH)D_3_, and 25(OH)D_3_ cellular concentrations at t0 were 1.12, 3.77, and 1.89 µM, respectively (*n* = 3). Bars with unlike letters were significantly different for a given variable.

As shown in Figure 3a,b, the vitamin D forms displayed significantly different uptake efficiencies in Caco-2 cell monolayers at high and low initial concentrations, respectively. D_3_ showed the lowest uptake rate (18% at high concentration, i.e., >5 µM, and 10% at low concentration, i.e., <1 µM), as compared to 1α(OH)D_3_ (25% at high concentration, and 18% at low concentration) and 25(OH)D_3_ (34% and 29% at high and low concentrations, respectively).

The efflux rates of the three forms ranged between 7% and 15% and were not proportional to their respective uptake rates (D_3_ > 25(OH)D_3_ > 1α(OH)D_3_; *p* < 0.05, Figure 3c).

### 3.3. Effect of Membrane Protein Inhibition on Vitamin D Uptake by Caco-2 Cells

#### 3.3.1. SR-BI Inhibition by BLT1

**Figure 4:**
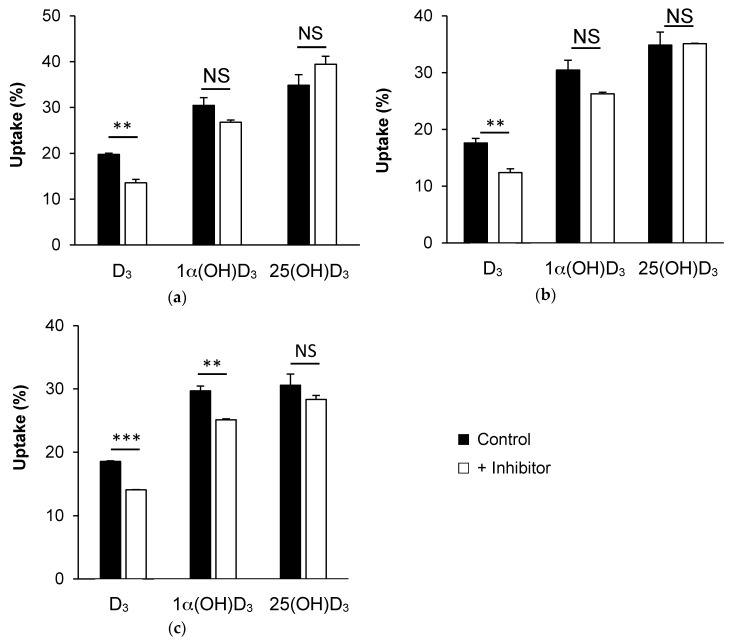
Uptake of vitamin D forms by Caco-2 cells in the presence of membrane protein inhibitors. Form (**a**) Uptake of D_3_, 1α(OH)D_3_, and 25(OH)D_3_ in the presence or absence of BLT1 (10 µM), an inhibitor of SR-BI (*n* = 3); (**b**) Uptake of D_3_, 1α(OH)D_3_, and 25(OH)D_3_ in the presence or absence of ezetimibe glucuronide (10 µM), an inhibitor of NPC1L1 (*n* = 3); (**c**) Uptake of D_3_, 1α(OH)D_3_, and 25(OH)D_3_ in the presence or absence of simvastatin (100 µM), an inhibitor of ASBT (*n* = 3). ** *p* < 0.01; *** *p* < 0.001, NS: not significant.

Figure 4a shows that the addition of 10 µM BLT1, the specific chemical inhibitor of SR-BI, significantly decreased D_3_ uptake from D_3_-enriched mixed-micelles (31%, *p* < 0.01) in Caco-2 cells. 1α(OH)D_3_ and 25(OH)D_3_ uptakes were not significantly modified in the presence of the inhibitor.

#### 3.3.2. NPC1L1 Inhibition by Ezetimibe Glucuronide

Similarly, Figure 4b shows that the addition of 10 µM of the specific chemical inhibitor of NPC1L1, i.e., ezetimibe glucuronide, significantly decreased D_3_ uptake (30%, *p* < 0.01), but not that of 1α(OH)D_3_ and 25(OH)D_3_.

#### 3.3.3. ASBT Inhibition by Simvastatin

Finally, Figure 4c shows that the addition of 100 µM simvastatin, which was shown to inhibit the membrane transporter ASBT [24], significantly (*p* < 0.01) decreased both D_3_ and 1α(OH)D_3_ uptake (about 24% and 15%, respectively), but not that of 25 (OH)D_3_.

### 3.4. Effect of ASBT Overexpression on D_3_ and 1α(OH)D_3_ Uptake by Griptite Cells

**Figure 5:**
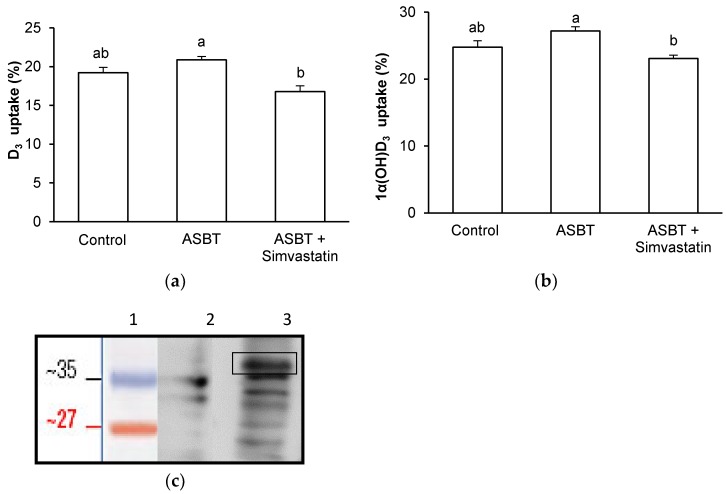
Effect of transfection with human ASBT (Apical Sodium-dependent Bile acid Transporter), and of simvastatin on uptake of vitamin D forms by Griptite cells. The cells were incubated with either D_3_; (**a**) or 1α(OH)D_3_; (**b**) +/− simvastatin (*n* = 3). Vitamin D form uptake was measured at 37 °C after 1 h incubation. Data are triplicates from one experiment representative of at least two independent experiments. Bars with unlike letters were significantly different for a given variable; (**c**) Western blots of ASBT expression in Griptite cells (1: Size Marker, 2: Griptite cells transfected with an empty plasmid, 3: Griptite cells overexpressing ASBT −38 KDa).

In order to confirm the involvement of ASBT in vitamin D form uptake, which was suggested by the inhibitory effect of simvastatin in Caco-2, we investigated the uptake of D_3_ and 1α(OH)D_3_ in Griptite cells overexpressing ASBT (Figure 5c). Figure 5a,b show respectively that ASBT overexpression changed neither D_3_ nor 1α(OH)D_3_ uptake.

## 4. Discussion

Because vitamin D status is generally not optimal in humans, supplementation solutions with either D_3_ or its bioactive forms may be necessary to restore patient status. To better understand the differences in biopotency of three vitamin D forms, namely D_3_, 25(OH)D_3_, and 1α(OH)D_3_, we thus compared their behavior during two key steps involved in their bioavailability, i.e., their incorporation into mixed micelles and their uptake by the enterocyte.

We first showed that the micellar incorporation of vitamin D forms was linear and ranked as follows: 1α(OH)D_3_ > D_3_ > 25(OH)D_3_. This result may be linked to the repartition of the hydroxyl groups in the molecules: they are grouped on one side for 1α(OH)D_3_, meaning it possesses well separated hydrophobic and hydrophilic sides, whereas they are located at both extremities for 25(OH)D_3_, thereby hindering its insertion into mixed micelles, which are composed of a hydrophobic core and a hydrophilic corona. 

In a second set of experiments, we showed that vitamin D uptake efficiency by Caco-2 cells ranked as follows: 25(OH)D_3_ > 1α(OH)D_3_ > D_3_, at both low and high concentrations. A close look at the results highlighted the fact that the three forms showed higher uptake rates at high concentrations, which is likely due to a higher contribution of passive diffusion through the brush border [9]. Our results suggest that vitamin D form uptake by the enterocyte is linked to their hydrophobicity (log P = 6.2, 6.8, and 7.9 for, respectively, values from PubChem Website). Indeed, this may affect their affinity for putative membrane proteins. Besides, the efflux of D_3_ from the cell compartment to the apical medium, which represents the intestinal lumen, was significantly more important than that of 25(OH)D_3_ and 1α(OH)D_3_. This result may be explained by the presence of a cytosolic vitamin D binding protein (cDBP) [25], which would display a higher affinity for hydroxylated forms than for D_3_, similarly to plasma DBP [26].

Overall, our in vitro results showed that the biopotencies (calculated as micellar incorporation × cell uptake) of D_3_, 25(OH)D_3_, and 1α(OH)D_3_ were 5.49 ± 0.55%, 6.67 ± 1.21%, and 13.19 ± 1.38%, respectively. 1α(OH)D_3_ biopotency was significantly higher than the biopotencies of the two other forms. These results are in agreement with a recent work that showed that the bioactivity of 1α(OH)D_3_ was higher than that of 25(OH)D_3_ in chicken [27].

In order to better understand the molecular mechanisms underlying vitamin D form uptake in Caco-2 cells, we explored the involvement in this phenomenon of three membrane proteins: SR-BI, NPC1L1 and ASBT. We specifically focused on these proteins because SR-BI and NPC1L1 have already been involved in vitamin D_3_ uptake in Caco-2 cells [9], and because SNPs in ASBT have been associated with the variability of D_3_ bioavailability in humans [11]. As expected, we confirmed the involvement of both SR-BI and NPC1L1 in D_3_ uptake by Caco-2 cell monolayers by using the inhibitors BLT1 and ezetimibe glucuronide, respectively. However, both inhibitors failed to significantly decrease 25(OH)D_3_ and 1α(OH)D_3_ uptake, highlighting the fact that these two molecules may be taken up by another pathway(s). We then investigated the involvement of the transporter ABST. The fact that ASBT could, even moderately, be involved in vitamin D uptake was hypothesized, as this transporter is involved in the uptake of bile salts, which display chemical structures close to that of vitamin D. Moreover, ASBT is mainly expressed in the ileum, which is consistent with the observation that a part of vitamin D is absorbed in the distal intestine [28]. We thus used simvastatin as a proposed specific inhibitor in acute condition, as previously suggested [24]. Both D_3_ and 1α(OH)D_3_ uptake were significantly decreased in the presence of the statin in Caco-2 cells, while 25(OH)D_3_ uptake remained unchanged. The finding that D_3_ and 1α(OH)D_3_ uptake was inhibited by this statin could be taken as initial evidence that ASBT is involved in the uptake of these forms. However, this result was not confirmed in Griptite cells transfected with ASBT. To explain this apparent discrepancy, we suggest that this statin is actually not a specific inhibitor of ASBT. We thus suggest that ASBT involvement in vitamin D form uptake is minor in vitro.

## 5. Conclusions

On the one hand, we showed that conversely to D_3_, 1α(OH)D_3_ and 25(OH)D_3_ uptake was neither SR-BI- nor NPC1L1-dependent. Besides, ASBT was not involved, at least in vitro, in the uptake of any of the three vitamin D forms. On the other hand, our data highlighted that 1α(OH)D_3_ displayed a higher ability to be absorbed by the intestine than D_3_ and 25(OH)D_3_. These results can partly explain the previously described high biopotency of 1α(OH)D_3_ compared to other forms. This work highlights the potential interest of using this molecule in the treatment of severe vitamin D deficiency.
